# The essential mass transfer step in hierarchical/nano zeolite: surface diffusion

**DOI:** 10.1093/nsr/nwz208

**Published:** 2019-12-20

**Authors:** Jian Zhou, Wei Fan, Yangdong Wang, Zaiku Xie

**Affiliations:** State Key Laboratory of Green Chemical Engineering and Industrial Catalysis, Shanghai Research Institute of Petrochemical Technology, SINOPEC, China; Department of Chemical Engineering, University of Massachusetts Amherst, USA; State Key Laboratory of Green Chemical Engineering and Industrial Catalysis, Shanghai Research Institute of Petrochemical Technology, SINOPEC, China; State Key Laboratory of Green Chemical Engineering and Industrial Catalysis, Shanghai Research Institute of Petrochemical Technology, SINOPEC, China; SINOPEC, China

An important topic in zeolite catalyst design is how to improve utilization efficiency of active sites in micropores by upgrading the effective diffusivity of reactants/products in zeolite crystal particles [1]. Nano- and hierarchical structures have drawn lots of attention in zeolitic catalysis fields in recent years, because these two methods can obviously improve the catalysis efficiency by shortening the configuration diffusion length [2]. However, new problems were raised in the construction process of hierarchical or nano-zeolites. For instance, it was found that the hierarchical structure can change the intrinsic properties of surface acid sites in zeolite [3]. Fan *et al*. reported that when the length scale of the zeolite approaches several nanometers, the diffusional time constants [4,5] and the apparent configuration diffusion lengths will be significantly increased than expected [6]. Therefore, enhanced mass transport in hierarchical zeolite will be overpredicted if not considering the extent of sorbate-sorbent interaction. Lercher's group also found that the rate determining step of the transport processes depends on the particle size [7]. As the common point in these reports, the surface barrier, also known as the resistance in surface diffusion, increases obviously in hierarchical or nano-zeolites and becomes an important factor influencing the overall diffusion process [6,8]. Increased resistance in surface diffusion means that the surface diffusion step becomes essential over whether hierarchical or nano-zeolites. Herein, for better understanding of this process, the behavioral characteristics and the action mechanism of surface diffusion, which are different from that of the traditional configuration, Knudsen or molecular diffusion process, are summarized. The future development and some possible applications of surface diffusion are also discussed.

Firstly, the surface diffusion of molecules in zeolites is a kind of transport process on surface instead of the collision with zeolitic surface, which is similar as the configuration diffusion. But the difference between them is that the former usually occurs in the mesopores, while the latter occurs in the micropores. Another difference is that sorbent molecules can be impacted by the micropore walls from all directions in the configuration diffusion, but the surface diffusion is usually only influenced by the molecular adsorption side. The interactions of guests with no matter the micropore wall or the external surface can be quantified by the adsorption enthalpy which is usually determined by the characteristics of guest molecules and the surface acidity. Generally, the type and status of species on surface are important factors influencing the surface diffusion, but the configuration diffusion is only controlled by the configuration of molecules and the limitation degree of micropores. The non-uniformity of the zeolite surface, such as the surface defect, the surface coverage and the pore-mouth blockage, is another key factor influencing the transport of molecules adsorbed on the surface. However, the impact of non-uniformity of the zeolite surface on surface diffusion still needs further development of quantitative characterization of the external surface structures. Moreover, the surface diffusion process usually needs an activation process, although its activation energy is lower than that of the configuration diffusion. In contrast, activation is not needed in Knudsen diffusion process. The characteristics details of different kinds of diffusion are listed in Table 1.

**Table 1.** Basic characteristics of four different kinds of diffusion in hierarchical/nano-zeolite.

Type	Activation energy	Function of surface in transport	Occurrence location	Impact on catalysis	Quantitative law
Molecular diffusion	-	-	Inside macropores	-	Fick's law
Knudsen diffusion	No activation process	Surface collision	Inside mesopores	Influencing utilization efficiency	Fick's law
Surface diffusion	Low	Surface transport	Surface of mesopores	Influencing utilization efficiency	-
Configuration diffusion	High	Surface transport	Inside micropores	Influencing selectivity of products	-

The surface diffusion is closely related to the surface characteristics. As the prior step, sorbent molecules should be absorbed on the surface of zeolites. If the adsorption strength is too strong, it will be difficult for the sorbent molecule to leave the surface. The transport process on the external surface will tend to be followed by re-entering of diffusing molecules into the micropores. The apparent diffusion lengths will thereby be significantly longer than predicted. As mentioned in the reports [6,8], the surface barrier in diffusion obviously increases in hierarchical or nano-zeolites, indicating much stronger interaction between the external surface and the sorbent molecules which consequently will lead to much longer apparent diffusion length [5]. The increase of non-uniformity of surface in hierarchical/nano zeolites is a major cause for their slow surface diffusion. The property of sorbent molecules is another factor influencing surface diffusion. For example, the aromatics and bulky molecules are dominant for the surface diffusion rather than small paraffin and olefin molecules. Recently, a fitting approach was developed by Liu's group to directly quantify surface barriers and it was found that the surface permeability is sensitive to the physical and chemical properties of the crystalline surface and the host-guest interaction at the surface [9]. Moreover, because the adsorption strength between sorbent molecules and zeolitic surface is closely related to the status of surface species, the concentration gradient is not the only driving force of surface diffusion, which means the Fick's law is not applicable in this process.

In general, because of the increase of the external acids and non-uniformity of surface in hierarchical/nano zeolites, the contribution of interaction of guest molecules with the external surface on the overall transport process is remarkably enhanced, which leads to higher probability of surface diffusion. In that case, the surface diffusion cannot be completely avoided; however, some measures such as appropriate surface modifications should be effective to decrease the impact of severe surface diffusion which is common in hierarchical/nano zeolites. The aim of modifications is to decrease the adsorption strength or to reduce the probability to re-enter into micropores and repeat the micropore diffusion process. The modification usually refers to the covering (Fig. 1) or the overlayer of inert species, which can moderate the zeolite surface properties [10,11]. Moreover, in the shaping process of practical catalysts, the binders are indispensably employed to satisfy necessary mechanical strength requirement for the industrial applications. As catalytic inert components, the addition of binders can also reduce the surface diffusion in zeolites. Although these measures for surface modifications may also lead to some problems, such as pore blockage and intercrystalline space occupation, the overall mass transport process might be still benefited because a better combined diffusion balance point can be reached, which should draw the future research attention for designing practical industrial hierarchical/nano zeolite catalysts. On the other hand, surface diffusion does not only play the negative role in the catalytic process; it can also be utilized for improving the selectivity. Although the shape selectivity and reaction path selectivity are respectively two frequently-used methods for improving the selectivity of products in zeolitic catalytic process, utilizing the surface diffusion properties of different molecules for higher selectivity of target products should be another research point in hierarchical/nano zeolite catalyst designing. Moreover, the surface diffusion under a relatively high concentration close to reaction conditions is another research field to which close attention should be paid, because it reflects real reactions over practical zeolite catalysts. Therefore, in-situ methods under reaction circumstance, including spectroscopic, gravity and concentration characterization technologies, or even synergistic tools, should be developed for investigating the surface diffusion behavior. The probe reaction, such as cracking of bulky molecules, is a meaningful tool for surface diffusion research which can be employed as the standard to quantify the surface diffusion.

**Figure 1.**
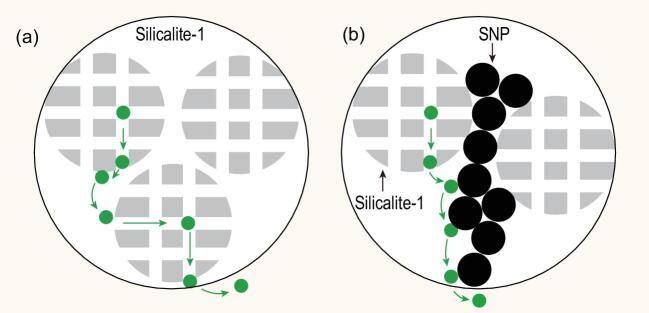
Proposed diffusion pathway of guest molecules in nano-zeolite (a) and the impact of surface covering with small silica particles (b) [11]. Copyright 2018, American Chemical Society Publications.

## References

[1] Shi J , Wang Y, Yang W et al. Chem Soc Rev 2015; 44: 8877–903. 26567526 10.1039/c5cs00626k

[2] Pérez-Ramírez J , Christensen CH, Egeblad K et al. Chem Soc Rev 2008; 37: 2530–42. 18949124 10.1039/b809030k

[3] Zhou J , Liu Z, Li L et al. Chin J Catal 2013; 34: 1429–33.

[4] Chang C-C , Teixeira AR, Li C et al. Langmuir 2013; 29: 13943–50. 24099522 10.1021/la403706r

[5] Vattipalli V , Qi X, Dauenhauer PJ et al. Chem Mater 2016; 28: 7852–63.

[6] Teixeira AR , Chang C-C, Coogan T et al. J Phys Chem C 2013; 117: 25545–55.

[7] Gobin OC , Reitmeier SJ, Jentys A et al. J Phys Chem C 2009; 113: 20435–44.

[8] Zhang L , Chmelik C, van Laak ANC et al. Chem Commun 2009; 45: 6424–6. 10.1039/b914391b 19841797

[9] Gao M , Li H, Yang M et al. Commun Chem 2019; 2: 43.

[10] Reitmeier SJ , Gobin OC, Jentys A et al. Angew Chem Int Ed 2009; 48: 533–8. 10.1002/anie.200803869 19072809

[11] Qi X , Vattipalli V, Dauenhauer PJ et al. Chem Mater 2018; 30: 2353–61.

